# Elastic Band Causing Exfoliation of the Upper Permanent Central Incisors

**DOI:** 10.1155/2015/186945

**Published:** 2015-03-22

**Authors:** Monica Ghislaine Oliveira Alves, Dárcio Kitakawa, Joao Batista Macedo Becker, Adriana Aigotti Haberbeck Brandão, Luiz Antonio Guimarães Cabral, Janete Dias Almeida

**Affiliations:** ^1^Departamento de Biociências e Diagnóstico Oral, Instituto de Ciência e Tecnologia, Universidade Estadual Paulista (UNESP), Avenida 777 Engenheiro Francisco José Longo, Jardim São Dimas, 12245-000 São José dos Campos, SP, Brazil; ^2^Departamento de Ciências Sociais e Odontopediatria, Instituto de Ciência e Tecnologia, Universidade Estadual Paulista (UNESP), Avenida 777 Engenheiro Francisco José Longo, Jardim São Dimas, 12245-000 São José dos Campos, SP, Brazil

## Abstract

*Objective*. This study reports a case in which elastic band use culminated in the loss of the incisors. *Case Report*. An 11-year-old white girl was seen complaining of pain, with purulent discharge and severe tooth mobility. The bone destruction detected radiographically in the region, despite its single location and absence in posterior quadrants of the maxilla and/or mandible, was similar to that observed in Langerhans cell disease. To our surprise, an elastic band involving the midportion of the roots of the two upper central incisors was found during biopsy. The debris was removed and a metal wire was placed in permanent maxillary right and left incisors. The patient was followed up, but no improvement in tooth mobility was observed. Bone loss increased, and internal resorption and root exposure occurred, which culminated in the extraction of permanent maxillary right and left incisors. *Conclusion*. The present case highlights the fact that professionals sometimes are confronted by anamnestic reports never seen before.

## 1. Introduction

Elastic bands are frequently used in dentistry to close interproximal spaces, to realign teeth, and to provide intermaxillary fixation in cases of fractures or orthodontic treatment [1]. In the past, elastic bands were used for intentional exfoliation in hemophilic patients [2, 3]. However, deleterious effects resulting from the incorrect use of elastic bands in orthodontics have been reported in the dental literature [4–9].

Controversy exists regarding the direct placement of an elastic band around a tooth in order to close diastema between incisors, to correct molar cross-bite, or to rotate poorly positioned teeth, since the elastic band is prone to migrate subgingivally along the root, causing destruction of the periodontal ligament and alveolar bone loss and subsequent exfoliation of the teeth involved [10].

This study reports a case in which elastic band use culminated in the loss of the upper permanent central incisors.

## 2. Case Report

**Figure 1:**
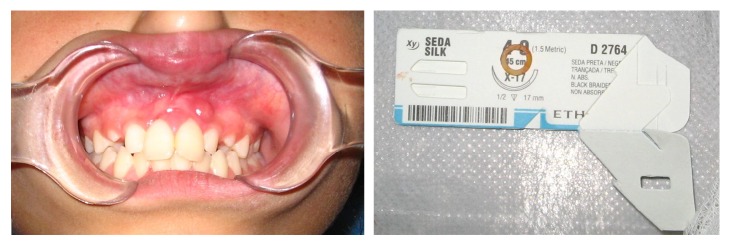
Initial clinical findings and elastic artifact.

An 11-year-old white girl with mixed dentition sought the stomatology outpatient clinic of our institution complaining of pain. The gums around permanent maxillary right and left incisors were compromised, including a purulent discharge and severe tooth mobility (Figure 1). During anamnesis, there was no mentioning or suspicion of systemic and/or hereditary diseases, deleterious habits, or local injury.

**Figure 2:**
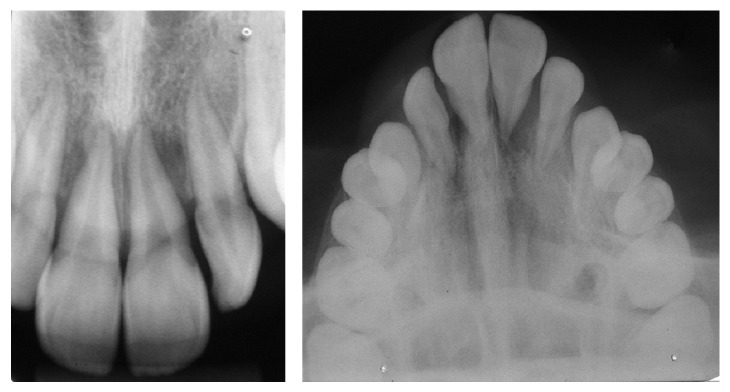
Initial radiographic findings.

Radiography revealed severe vertical bone loss in both upper incisors (Figure 2), which did not respond to electric or thermal pulp vitality tests. The diagnosis of an odontogenic abscess was ruled out due to the absence of trauma or caries in the teeth involved. However, the single location of the condition, its absence in posterior quadrants of the maxilla and/or mandible, its exudative appearance, and the radiographic demonstration of bone destruction were similar to the findings seen in Langerhans cell disease.

**Figure 3:**
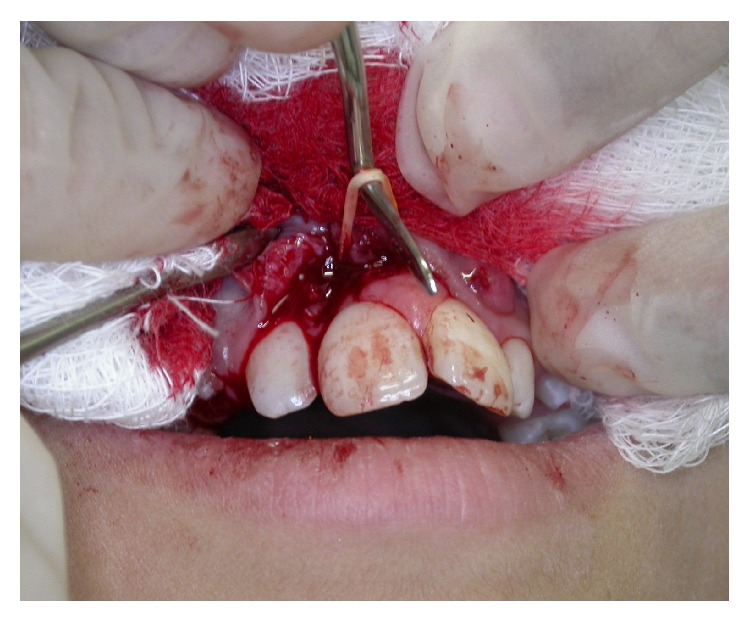
Transoperative clinical findings.

To our surprise, an elastic band involving the midportion of the roots of the two central incisors was found during biopsy (Figure 3). When asked about the presence of the elastic band, the patient responded that she had obtained the band from the hair of her doll and had placed it around her teeth because a dentist had treated a friend with diastema in the same region using an elastic band.

**Figure 4:**
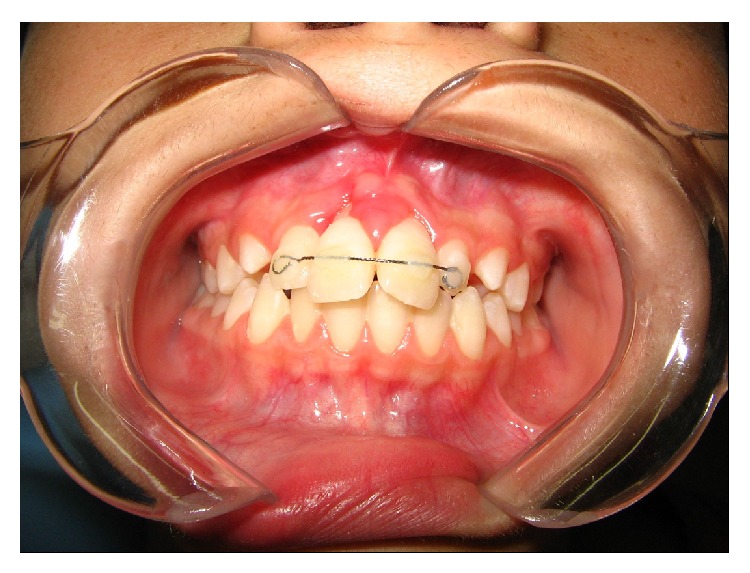
Postoperative clinical findings.

The debris was removed and a metal wire was placed in permanent maxillary right and left incisors (Figure 4). In addition, exfoliative cytology was performed which demonstrated a large number of neutrophils, macrophages, and red blood cells, few lymphocytes, sparse pavement epithelial cells with inflammatory alterations, filamentous mucus, and cell remnants. There were no signs of malignancy. The cytological diagnosis was class II Papanicolaou compatible with an acute inflammatory process.

The patient was followed up by clinical and radiographic examination, but no improvement in tooth mobility was observed. Bone loss increased and internal resorption and root exposure occurred. As a consequence, permanent maxillary right and left incisors were extracted after 2 months. The patient was referred for orthodontic treatment which consisted of a removable Hawley style retainer with an expansion screw in the middle for functional and esthetic improvement.

## 3. Discussion

Several studies have alerted to the harmful effects of the incorrect use of orthodontic elastic bands [4–9]. Placement of orthodontic elastic bands around the cervical part of teeth can cause localized periodontal destruction by subgingival migration of the band and subsequent tooth loss [4]. In a literature review, Huang and Creath (1995) concluded that the direct application of an orthodontic elastic band around teeth without associated orthodontic braces to close maxillary anterior diastema is contraindicated [11].

Other investigators recommend a series of precautions to prevent common complications associated with elastic band use. In this respect, elastics should not be used around the crowns of teeth without stabilization through brackets or directly bonded to the teeth. The professional should be aware of the occurrence of subgingival migration, which can cause pain and/or gingival inflammation. It is also important to explain the risks to the patient and family. Furthermore, radiopaque material should be incorporated in orthodontic elastic bands to facilitate radiographic localization [10]. In the present case, the elastic band was not a true orthodontic band. According to the patient, she had removed the elastic band from the hair of her doll and had placed it around her teeth because a dentist had treated a friend with diastema in the same region using an elastic band.

To our knowledge, this is the first case wherein the rubber band was placed by the patient instead of dentists in the English literature. The iatrogenic loss of a clinical attachment level caused by interdental rubber bands placed by dentists was first described in 1870 [12]. Sánchez-Pérez et al. (2006) reported a case of a nine-year-old boy presenting a serious periodontal lesion caused by a rubber band that had been used to close a medial diastema. The authors concluded that impaction of a foreign body in the periodontal space should be suspected when a clinical attachment level is lost during childhood [13].

Lin et al. (2011) emphasized that patients are generally aware of the relationship between injury and elastic artifacts. The authors reported three cases in which the improper use of orthodontic elastic bands to close maxillary anterior diastema caused the formation of pyogenic-like granulomas in the interdental area. Common signs and symptoms were a painful sensation, aggressive periodontal destruction leading to the formation of large periodontal pockets, prominent tooth extrusion, and severe tooth mobility, similar to the findings observed in the present case [1].

Finkbeiner et al. (1997) concluded that the clinician should monitor periodontal features when elastic bands are used and that patients should be advised not to perform self-treatment since the outcome can be disastrous as in the present case [14].

The key treatment for this iatrogenic trauma consists of surgical removal of the elastic bands and debris or a nonsurgical periodontal approach, depending on the depth of the artifact and severity of periodontal destruction [1].

Papanicolaou method evaluates changes in epithelial tissues [13]; in this case, the debris was removed and exfoliative cytology was performed to reaffirm the acute inflammatory characteristic of the lesion, which was clearly caused by the presence of the foreign body.

The present case highlights the importance of thorough assessment of the patient by accurate anamnesis and physical examination, in order to check the points that were not mentioned by the patient.
